# Supplementary material for: A fast machine-learning-guided primer design pipeline for selective whole genome amplification
Source: PLoS Comput Biol. 2023 Apr 17;19(4):e1010137. doi: 10.1371/journal.pcbi.1010137 (PMC10138271; doi:10.1371/journal.pcbi.1010137)
Supplement: S1 Appendix — (PDF) [file pcbi.1010137.s001.pdf]

S1 Appendix

Algorithm 1: Primer Set Search

```
Input: primer_list (list of candidate primers)
       max_sets (maximum number of sets to explore at each stage)
       amp_efficacy_scores (amplification efficacy scores predicted from random forest
   model)
       drop_indices (defines which iterations are drop-out layers)
Output: top_sets
top_sets ← RandomInitialStart (primer_list, amp_efficacy_scores, max_sets);
top_scores ← Evaluate (top_sets);
curr_sets ← [];
curr_scores ← [];
for i = 1 to max_iterations do
    for top_set in top_sets do
        for primer in S do
            if Compatible (top_set ∪ [primer]) then
                new_set ← [top_set ∪ [primer]];
                score ← Evaluate (new_set);
                curr_scores ← curr_scores ∪ [score];
                curr_sets ← curr_sets ∪ new_set;
            end
        end
    end
    if max(curr_scores) < min(top_scores) + ϵ then
        return top_sets
    end
    if i is in drop_indices then
        top_sets, top_scores = DropOut (curr_scores, curr_sets, max_sets)
    end
    else
        top_sets, top_scores = ChooseMaxSets (curr_scores, curr_sets, max_sets)
    end
end
return top_sets
```
