# Supplementary material for: A fast machine-learning-guided primer design pipeline for selective whole genome amplification
Source: PLoS Comput Biol. 2023 Apr 17;19(4):e1010137. doi: 10.1371/journal.pcbi.1010137 (PMC10138271; doi:10.1371/journal.pcbi.1010137)
Supplement: S1 Table — (PDF) [file pcbi.1010137.s002.pdf]

S1 Table: S1 Table: Primer Amplification Data

Round	primer name	Primer sequence	Primer molarity	target	pcDNA.1	pcDNA.2	pcDNA.3	pLTR.1	pLTR.2	pLTR.3	Mean Amplification for pcDNA	Standard deviation for pcDNA	Mean Amplification for pLTR	Standard deviation for pLTR
1	pcDNA_24	ACAACC	0.2mM	pcDNA	0.73	0.72	1.37	0.65	0.65	0.91	0.94	0.37	0.74	0.15
1	pcDNA_41	GAATCT	0.2mM	pcDNA	0.75	0.59	2.97	0.65	0.55	1.64	1.44	1.33	0.95	0.60
1	pcDNA_45	CGCACT	0.2mM	pcDNA	0.67	0.73	3.41	0.64	0.79	3.69	1.60	1.57	1.71	1.72
1	pcDNA_67	ATGGAC	0.2mM	pcDNA	2.60	1.88	11.50	2.30	2.25	7.70	5.33	5.36	4.08	3.13
1	pcDNA_77	ATCAAC	0.2mM	pcDNA	1.08	0.74	4.70	0.89	0.70	1.92	2.17	2.20	1.17	0.66
1	pcDNA_83	ATTTGC	0.2mM	pcDNA	2.93	1.75	12.80	3.22	1.20	8.27	5.83	6.07	4.23	3.64
1	pcDNA_94	TCGCGG	0.2mM	pcDNA	3.91	3.12	2.74	2.11	3.26	1.42	3.26	0.60	2.26	0.93
1	pcDNA_96	GGCCCGT	0.2mM	pcDNA	6.49	1.72	2.51	1.94	3.21	1.69	3.57	2.56	2.28	0.82
1	pcDNA_806	GTTATGG	0.2mM	pcDNA	9.64	10.00	12.50	5.03	7.81	6.11	10.71	1.56	6.32	1.40
1	pcDNA_1090	GGTCGAC	0.2mM	pcDNA	25.30	16.80	25.00	13.70	20.00	11.30	22.37	4.82	15.00	4.49
1	pcDNA_1152	ACCCCCA	0.2mM	pcDNA	14.10	9.63	13.80	7.11	9.28	5.72	12.51	2.50	7.37	1.79
1	pcDNA_1269	AACATTT	0.2mM	pcDNA	0.76	0.57	0.68	0.73	0.90	0.76	0.67	0.10	0.80	0.09
1	pcDNA_1278	GGCCGAC	0.2mM	pcDNA	12.70	10.60	42.20	4.47	4.15	24.50	21.83	17.67	11.04	11.66
1	pcDNA_1683	GGGGCAC	0.2mM	pcDNA	26.40	19.30	46.10	16.80	16.60	43.60	30.60	13.88	25.67	15.53
1	pcDNA_2024	ATGAAGCC	0.2mM	pcDNA	5.65	3.87	10.30	2.40	2.50	27.30	6.61	3.32	10.73	14.35
1	pcDNA_2464	ACTCCACC	0.2mM	pcDNA	2.54	2.25	1.15	1.27	3.02	1.09	1.98	0.73	1.79	1.07
1	pcDNA_2491	AATGTCAA	0.2mM	pcDNA	17.60	7.93	24.40	14.50	8.08	13.70	16.64	8.28	12.09	3.50
1	pcDNA_4525	AACAAGGC	0.2mM	pcDNA	37.30	19.80	55.00	26.50	20.20	58.00	37.37	17.60	34.90	20.25
1	pcDNA_5268	GCAACTTT	0.2mM	pcDNA	0.78	0.82	0.72	0.81	1.08	0.58	0.77	0.05	0.83	0.25
1	pcDNA_5476	GTGCCAAA	0.2mM	pcDNA	15.00	12.20	26.30	18.30	19.20	19.70	17.83	7.46	19.07	0.71
1	pcDNA_6421	GTCGCCGT	0.2mM	pcDNA	7.66	5.99	12.30	9.98	7.35	4.74	8.65	3.27	7.36	2.62
1	pcDNA_6570	GGAAATCCC	0.2mM	pcDNA	10.30	4.32	4.73	9.49	6.25	4.17	6.45	3.34	6.64	2.68
1	pcDNA_6855	AATAATCAA	0.2mM	pcDNA	23.40	24.70	34.40	15.20	23.20	22.40	27.50	6.01	20.27	4.41
1	pcDNA_6861	TTCTTCGGG	0.2mM	pcDNA	13.10	11.10	20.60	10.20	12.20	13.70	14.93	5.01	12.03	1.76
1	pcDNA_7313	GGAAGGCAC	0.2mM	pcDNA	19.80	19.80	40.90	12.90	13.60	42.70	26.83	12.18	23.07	17.01
1	pcDNA_8555	GGGGGAGGA	0.2mM	pcDNA	156.00	116.00	212.00	141.60	164.60	228.00	161.33	48.22	178.07	44.75
1	pcDNA_10992	AACTGCCCA	0.2mM	pcDNA	7.46	5.73	8.37	3.84	19.40	5.97	7.19	1.34	9.74	8.44
1	pcDNA_11280	TTCAAGGAG	0.2mM	pcDNA	8.39	6.27	15.80	11.60	12.60	27.10	10.15	5.00	17.10	8.67
1	pcDNA_12917	CCGCCATGCC	0.2mM	pcDNA	19.30	11.10	19.60	195.50	157.90	39.50	16.67	4.82	130.97	81.41
1	pcDNA_13523	TTGCCTTGTT	0.2mM	pcDNA	9.25	5.87	45.50	8.01	7.71	12.90	20.21	21.97	9.54	2.91
1	pcDNA_14323	GCTCTAGGGG	0.2mM	pcDNA	81.40	70.00	97.20	59.80	61.20	73.00	82.87	13.66	64.67	7.25
1	pcDNA_15207	GGCTGGCTGG	0.2mM	pcDNA	59.00	42.90	72.40	54.00	53.00	57.00	58.10	14.77	54.67	2.08
1	pcDNA_16011	GGGATAATAC	0.2mM	pcDNA	22.40	15.30	29.30	25.30	17.00	22.10	22.33	7.00	21.47	4.19
1	pcDNA_16881	AGGTTTCCCG	0.2mM	pcDNA	59.70	25.80	21.80	17.30	7.29	4.20	35.77	20.82	9.60	6.85
1	pcDNA_17849	TTCGCCAGTT	0.2mM	pcDNA	3.76	4.00	3.09	3.34	3.55	2.14	3.62	0.47	3.01	0.76
1	pcDNA_19424	TGCCAAAACAA	0.2mM	pcDNA	14.80	14.00	10.40	20.60	28.40	5.77	13.07	2.34	18.26	11.50
1	pcDNA_20317	CCAGTCCGCCC	0.2mM	pcDNA	8.34	8.72	12.60	3.17	4.37	3.87	9.89	2.36	3.80	0.60
1	pcDNA_20438	CTCATTTTATT	0.2mM	pcDNA	17.90	19.60	20.30	20.90	19.50	19.20	19.27	1.23	19.87	0.91
1	pcDNA_20875	ACGTCATTATT	0.2mM	pcDNA	13.50	7.09	9.89	10.10	11.10	4.27	10.16	3.21	8.49	3.69
1	pcDNA_21088	GGGGGAGGGGC	0.2mM	pcDNA	93.20	74.80	80.20	59.20	91.60	67.20	82.73	9.46	72.67	16.88

target = Plasmid with exact match for primer

pcDNA.1 = Post-amplification concentration (ng/uL) of DNA from plasmid pcDNA in the 1st experimental replicate. Concentration measued using the Qubit dsDNA HS Assay Kit (Thermo Fisher Scientific)

S1 Table: Primer Amplification Data (continued)

Round	primer name	Primer sequence	Primer molarity	target	pcDNA.1	pcDNA.2	pcDNA.3	pLTR.1	pLTR.2	pLTR.3	Mean Amplification for pcDNA	Standard deviation for pcDNA	Mean Amplification for pLTR	Standard deviation for pLTR
1	pcDNA_22656	CGCCACAACAT	0.2mM	pcDNA	27.90	9.58	19.60	120.20	40.50	6.44	19.03	9.17	55.71	58.39
1	pcDNA_24137	GAACAGCTCCT	0.2mM	pcDNA	10.20	3.57	5.71	5.38	6.00	4.09	6.49	3.38	5.16	0.97
1	pcDNA_25527	ACTTACGGTAAA	0.2mM	pcDNA	32.60	44.60	28.60	27.00	30.70	12.90	35.27	8.33	23.53	9.39
1	pcDNA_25881	CGGTAAGATCCT	0.2mM	pcDNA	7.16	5.03	6.97	3.46	3.65	2.91	6.39	1.18	3.34	0.38
1	pcDNA_26948	GAAGCGGAAGAG	0.2mM	pcDNA	61.40	27.40	64.70	54.40	42.80	45.30	51.17	20.65	47.50	6.10
1	pcDNA_30183	CAACGCGTATAT	0.2mM	pcDNA	42.10	90.60	58.00	28.50	33.60	38.80	63.57	24.72	33.63	5.15
1	pcDNA_30638	TGGGGTGGGGCA	0.2mM	pcDNA	79.60	80.10	110.70	38.60	58.00	57.00	90.13	17.81	51.20	10.92
1	pcDNA_31133	GTTTGCGCAACG	0.2mM	pcDNA	35.10	40.10	47.10	38.60	56.00	22.90	40.77	6.03	39.17	16.56
1	pLTR_3	GTGTTT	0.2mM	pLTR	0.97	0.83	3.40	0.99	0.91	3.40	1.73	1.45	1.77	1.41
1	pLTR_28	TTAAAC	0.2mM	pLTR	0.53	0.50	1.63	0.71	0.60	2.31	0.89	0.64	1.20	0.96
1	pLTR_46	TGAGGG	0.2mM	pLTR	2.78	2.10	14.10	2.56	2.40	8.86	6.33	6.74	4.61	3.68
1	pLTR_76	ATAGGA	0.2mM	pLTR	8.12	3.41	21.80	8.06	5.15	12.40	11.11	9.55	8.54	3.65
1	pLTR_78	TCGAGT	0.2mM	pLTR	0.96	0.91	3.12	0.79	0.98	2.39	1.66	1.26	1.38	0.88
1	pLTR_101	CTCGCA	0.2mM	pLTR	1.60	1.05	4.68	1.47	1.28	3.44	2.44	1.96	2.06	1.20
1	pLTR_115	TTACAG	0.2mM	pLTR	0.79	0.64	0.53	0.81	1.20	0.63	0.65	0.13	0.88	0.29
1	pLTR_325	GTCATCC	0.2mM	pLTR	1.67	0.89	1.55	1.48	1.71	0.83	1.37	0.42	1.34	0.45
1	pLTR_691	TCGGGCC	0.2mM	pLTR	6.08	2.51	7.27	2.85	2.87	1.58	5.29	2.48	2.43	0.74
1	pLTR_695	TGGGGAA	0.2mM	pLTR	25.70	12.90	42.20	37.40	21.30	35.40	26.93	14.69	31.37	8.78
1	pLTR_957	CCGGTCC	0.2mM	pLTR	2.12	1.75	1.88	2.61	3.53	1.58	1.92	0.19	2.57	0.98
1	pLTR_1165	ACCAACC	0.2mM	pLTR	1.95	1.58	2.21	1.44	2.85	0.89	1.91	0.32	1.73	1.01
1	pLTR_1766	AGACCTA	0.2mM	pLTR	1.64	0.84	4.68	1.55	1.31	9.51	2.39	2.02	4.12	4.67
1	pLTR_1869	TGGTTGG	0.2mM	pLTR	5.29	4.61	17.80	4.03	2.98	14.10	9.23	7.43	7.04	6.14
1	pLTR_2820	TAACAAGT	0.2mM	pLTR	1.41	1.04	5.27	1.71	1.50	4.17	2.57	2.34	2.46	1.48
1	pLTR_4412	GACCCCCC	0.2mM	pLTR	5.74	3.74	7.25	0.50	3.40	1.19	5.58	1.76	1.70	1.51
1	pLTR_5739	GTTTCAGT	0.2mM	pLTR	3.24	1.49	19.60	3.02	2.80	10.30	8.11	9.99	5.37	4.27
1	pLTR_5841	CCAGAACT	0.2mM	pLTR	1.97	1.16	5.40	1.94	1.45	3.40	2.84	2.25	2.26	1.01
1	pLTR_5941	ATACCACT	0.2mM	pLTR	1.38	1.89	1.96	1.28	2.05	1.92	1.74	0.32	1.75	0.41
1	pLTR_6164	GGTGGAGG	0.2mM	pLTR	41.80	30.90	71.20	48.40	58.00	74.20	47.97	20.85	60.20	13.04
1	pLTR_7002	TCATTTTGG	0.2mM	pLTR	18.60	9.08	17.30	17.40	12.40	13.90	14.99	5.16	14.57	2.57
1	pLTR_7658	CTAACGAGT	0.2mM	pLTR	7.23	5.09	8.75	8.88	6.08	9.03	7.02	1.84	8.00	1.66
1	pLTR_9278	AAGTAAAAC	0.2mM	pLTR	19.10	16.70	24.80	13.60	20.50	17.80	20.20	4.16	17.30	3.48
1	pLTR_9649	GTTTGACAG	0.2mM	pLTR	19.20	23.00	36.20	15.50	17.70	16.00	26.13	8.92	16.40	1.15
1	pLTR_10496	CTAAACGAG	0.2mM	pLTR	12.90	10.00	28.60	10.30	21.50	23.90	17.17	10.01	18.57	7.26
1	pLTR_11404	CCCACCCTA	0.2mM	pLTR	3.57	2.37	4.23	2.10	4.01	2.92	3.39	0.94	3.01	0.96
1	pLTR_12098	GGAAGGAAC	0.2mM	pLTR	58.60	55.00	82.80	35.50	48.50	58.00	65.47	15.12	47.33	11.30
1	pLTR_13226	TATTTTAAGC	0.2mM	pLTR	17.00	21.80	11.70	10.40	27.00	14.10	16.83	5.05	17.17	8.71
1	pLTR_13474	GTTTGACAGC	0.2mM	pLTR	40.60	45.20	74.40	18.90	43.00	59.00	53.40	18.33	40.30	20.19
1	pLTR_13738	TTTAAAAAAC	0.2mM	pLTR	24.70	18.40	33.90	17.60	18.00	24.40	25.67	7.80	20.00	3.82
1	pLTR_14165	GGCTAGAACA	0.2mM	pLTR	58.00	49.90	64.90	42.90	41.40	42.70	57.60	7.51	42.33	0.81
1	pLTR_16201	TCGTCAAACC	0.2mM	pLTR	3.98	2.76	3.50	3.45	2.76	3.35	3.41	0.61	3.19	0.37

target = Plasmid with exact match for primer
pcDNA.1 = Post-amplification concentration (ng/uL) of DNA from plasmid pcDNA in the 1st experimental replicate. Concentration measued using the Qubit dsDNA HS Assay Kit (Thermo Fisher Scientific)

S1 Table: Primer Amplification Data (continued)

Round	primer name	Primer sequence	Primer molarity	target	pcDNA.1	pcDNA.2	pcDNA.3	pLTR.1	pLTR.2	pLTR.3	Mean Amplification for pcDNA	Standard deviation for pcDNA	Mean Amplification for pLTR	Standard deviation for pLTR
1	pLTR_17265	CCTCCTCCCC	0.2mM	pLTR	0.81	0.74	0.71	2.08	0.84	0.68	0.76	0.05	1.20	0.77
1	pLTR_17397	ATTAGTTATT	0.2mM	pLTR	6.80	6.02	8.24	5.36	5.98	4.99	7.02	1.13	5.44	0.50
1	pLTR_19803	GGAGACTCCGT	0.2mM	pLTR	8.85	12.50	13.10	10.10	14.30	13.00	11.48	2.30	12.47	2.15
1	pLTR_19903	GGGGTCAGATA	0.2mM	pLTR	42.20	53.00	47.40	22.00	44.80	31.40	47.53	5.40	32.73	11.46
1	pLTR_19941	GGCCCCAATGG	0.2mM	pLTR	44.90	7.54	19.50	28.90	21.30	21.70	23.98	19.08	23.97	4.28
1	pLTR_20204	TTTTATGTTTC	0.2mM	pLTR	1.50	1.50	2.39	1.49	2.01	2.59	1.80	0.51	2.03	0.55
1	pLTR_20247	TTGTTGTTGTT	0.2mM	pLTR	23.70	14.20	13.30	26.00	27.20	7.99	17.07	5.76	20.40	10.76
1	pLTR_23461	TTGCTGGAGTG	0.2mM	pLTR	46.40	38.30	62.80	38.00	38.80	49.60	49.17	12.48	42.13	6.48
1	pLTR_26347	CCGTACCTCCT	0.2mM	pLTR	2.31	3.52	3.48	4.30	4.54	3.72	3.10	0.69	4.19	0.42
1	pLTR_26769	GCCCCTGTAACA	0.2mM	pLTR	5.06	4.49	7.45	4.92	6.02	6.60	5.67	1.57	5.85	0.85
1	pLTR_27475	CCTCTTCTACCT	0.2mM	pLTR	1.69	1.55	1.71	2.01	1.64	1.81	1.65	0.09	1.82	0.19
1	pLTR_27737	GGTGTGGGAGGT	0.2mM	pLTR	62.40	52.00	82.60	81.00	82.80	94.90	65.67	15.56	86.23	7.56
1	pLTR_29117	CCAGTCCCCCCT	0.2mM	pLTR	4.72	1.69	2.02	9.32	2.13	2.46	2.81	1.66	4.64	4.06
1	pLTR_29887	ATTGTTGTTGTT	0.2mM	pLTR	20.20	11.60	15.80	16.80	14.00	13.50	15.87	4.30	14.77	1.78
1	pLTR_30346	CAGGGCGAATAA	0.2mM	pLTR	89.70	89.20	137.20	66.40	100.50	96.50	105.37	27.57	87.80	18.64
1	pLTR_30742	CTGTGGAAATAA	0.2mM	pLTR	21.80	34.40	21.70	22.40	39.40	20.90	25.97	7.30	27.57	10.28
1	pcDNA_1278	GGCCGAC	0.5mM	pcDNA	1.87	0.46		0.55	0.17		1.17	0.99	0.36	0.27
1	pcDNA_12917	CCGCCATGCC	0.5mM	pcDNA	1.26			1.12			1.26	0.00	1.12	
1	pcDNA_13523	TTGCCTTGTT	0.5mM	pcDNA	0.65	0.68		0.91	0.40		0.66	0.02	0.65	0.36
1	pcDNA_14323	GCTCTAGGGG	0.5mM	pcDNA	7.53	4.67		7.04	4.31		6.10	2.02	5.68	1.93
1	pcDNA_1683	GGGGCAC	0.5mM	pcDNA	3.73	1.68		1.32	1.30		2.71	1.45	1.31	0.01
1	pcDNA_20317	CCAGTCCGCCC	0.5mM	pcDNA	7.30	2.86		1.62	1.12		5.08	3.14	1.37	0.35
1	pcDNA_21088	GGGGGAGGGGC	0.5mM	pcDNA	11.30	7.59		10.80	8.84		9.45	2.62	9.82	1.39
1	pcDNA_22656	CGCCACAACAT	0.5mM	pcDNA	2.36	0.73		1.16	0.37		1.54	1.15	0.76	0.56
1	pcDNA_30638	TGGGGTGGGGCA	0.5mM	pcDNA	29.00	19.70		14.20	11.40		24.35	6.58	12.80	1.98
1	pcDNA_5476	GTGCCAAA	0.5mM	pcDNA	0.72			0.36			0.72	0.00	0.36	
1	pcDNA_6855	AATAATCAA	0.5mM	pcDNA	0.76			0.31			0.76	0.00	0.31	
1	pcDNA_7313	GGAAGGCAC	0.5mM	pcDNA	4.35	1.33		2.13	1.11		2.84	2.14	1.62	0.72
1	pcDNA_8555	GGGGGAGGA	0.5mM	pcDNA	8.74	5.70		18.50	12.70		7.22	2.15	15.60	4.10
1	pLTR_10496	CTAAACGAG	0.5mM	pLTR	1.18	0.40		0.89	0.46		0.79	0.55	0.67	0.30
1	pLTR_12098	GGAAGGAAC	0.5mM	pLTR	3.25	1.62		3.49	2.28		2.44	1.15	2.89	0.86
1	pLTR_14165	GGCTAGAACA	0.5mM	pLTR	8.01	4.65		6.06	4.29		6.33	2.38	5.18	1.25
1	pLTR_1869	TGGTTGG	0.5mM	pLTR	1.12	0.44		0.53	0.40		0.78	0.48	0.46	0.09
1	pLTR_19903	GGGGTCAGATA	0.5mM	pLTR	25.70	12.00		17.10	7.61		18.85	9.69	12.36	6.71
1	pLTR_19941	GGCCCCAATGG	0.5mM	pLTR	13.50	6.15		5.87	6.08		9.83	5.20	5.98	0.15
1	pLTR_27737	GGTGTGGGAGGT	0.5mM	pLTR	17.70	15.20		23.80	30.70		16.45	1.77	27.25	4.88
1	pLTR_29117	CCAGTCCCCCCT	0.5mM	pLTR	0.51			0.98			0.51	0.00	0.98	
1	pLTR_29887	ATTGTTGTTGTT	0.5mM	pLTR	4.88			2.11			4.88	0.00	2.11	
1	pLTR_30742	CTGTGGAAATAA	0.5mM	pLTR	11.60	5.08		11.20	7.32		8.34	4.61	9.26	2.74
1	pLTR_6164	GGTGGAGG	0.5mM	pLTR	2.86	2.43		5.64	5.99		2.65	0.30	5.82	0.25
1	pLTR_695	TGGGGAA	0.5mM	pLTR	1.37			1.44			1.37	0.00	1.44	

target = Plasmid with exact match for primer

pcDNA.1 = Post-amplification concentration (ng/uL) of DNA from plasmid pcDNA in the 1st experimental replicate. Concentration measued using the Qubit dsDNA HS Assay Kit (Thermo Fisher Scientific)

S1 Table: Primer Amplification Data (continued)

Round	primer name	Primer sequence	Primer molarity	target	pcDNA.1	pcDNA.2	pcDNA.3	pLTR.1	pLTR.2	pLTR.3	Mean Amplification for pcDNA	Standard deviation for pcDNA	Mean Amplification for pLTR	Standard deviation for pLTR
1	pLTR_76	ATAGGA	0.5mM	pLTR	0.51			0.61			0.51	0.00	0.61	
1	pLTR_9649	GTTTGACAG	0.5mM	pLTR	0.64			0.49			0.64	0.00	0.49	
1	pcDNA_1278	GGCCGAC	1mM	pcDNA	1.03	0.87		0.66	0.31		0.95	0.12	0.49	0.24
1	pcDNA_12917	CCGCCATGCC	1mM	pcDNA	4.01			3.29			4.01	0.00	3.29	
1	pcDNA_13523	TTGCCTTGTT	1mM	pcDNA	1.15	0.91		1.36	1.02		1.03	0.17	1.19	0.24
1	pcDNA_14323	GCTCTAGGGG	1mM	pcDNA	21.40	18.80		14.40	13.30		20.10	1.84	13.85	0.78
1	pcDNA_1683	GGGGCAC	1mM	pcDNA	3.36	4.24		3.25	2.69		3.80	0.62	2.97	0.40
1	pcDNA_20317	CCAGTCCGCCC	1mM	pcDNA	3.83	3.30		1.56	1.43		3.57	0.37	1.50	0.09
1	pcDNA_21088	GGGGGAGGGGC	1mM	pcDNA	21.90	21.00		15.50	14.80		21.45	0.64	15.15	0.49
1	pcDNA_22656	CGCCACAACAT	1mM	pcDNA	7.15	3.90		3.35	0.87		5.53	2.30	2.11	1.75
1	pcDNA_30638	TGGGGTGGGGCA	1mM	pcDNA	40.70	44.40		21.40	18.00		42.55	2.62	19.70	2.40
1	pcDNA_5476	GTGCCAAA	1mM	pcDNA	1.08			0.77			1.08	0.00	0.77	
1	pcDNA_6855	AATAATCAA	1mM	pcDNA	3.39			1.41			3.39	0.00	1.41	
1	pcDNA_7313	GGAAGGCAC	1mM	pcDNA	3.35	5.13		3.52	3.45		4.24	1.26	3.49	0.05
1	pcDNA_8555	GGGGGAGGA	1mM	pcDNA	22.60	21.80		28.40	27.90		22.20	0.57	28.15	0.35
1	pLTR_10496	CTAAACGAG	1mM	pLTR	0.98	0.92		1.05	0.98		0.95	0.04	1.02	0.05
1	pLTR_12098	GGAAGGAAC	1mM	pLTR	11.10	9.25		8.26	7.99		10.18	1.31	8.13	0.19
1	pLTR_14165	GGCTAGAACA	1mM	pLTR	17.30	18.80		11.70	8.78		18.05	1.06	10.24	2.06
1	pLTR_1869	TGGTTGG	1mM	pLTR	0.76	0.64		0.76	0.57		0.70	0.09	0.66	0.13
1	pLTR_19903	GGGGTCAGATA	1mM	pLTR	35.80	29.90		21.60	16.90		32.85	4.17	19.25	3.32
1	pLTR_19941	GGCCCCAATGG	1mM	pLTR	9.67	11.40		9.26	11.20		10.54	1.22	10.23	1.37
1	pLTR_27737	GGTGTGGGAGGT	1mM	pLTR	45.30	55.00		56.00	47.90		50.15	6.86	51.95	5.73
1	pLTR_29117	CCAGTCCCCCCT	1mM	pLTR	1.09			1.79			1.09	0.00	1.79	
1	pLTR_29887	ATTGTTGTTGTT	1mM	pLTR	10.00			4.26			10.00	0.00	4.26	
1	pLTR_30742	CTGTGGAAATAA	1mM	pLTR	18.20	12.10		14.80	9.64		15.15	4.31	12.22	3.65
1	pLTR_6164	GGTGGAGG	1mM	pLTR	8.21	7.15		15.80	8.73		7.68	0.75	12.27	5.00
1	pLTR_695	TGGGGAA	1mM	pLTR	3.97			3.55			3.97	0.00	3.55	
1	pLTR_76	ATAGGA	1mM	pLTR	1.81			0.97			1.81	0.00	0.97	
1	pLTR_9649	GTTTGACAG	1mM	pLTR	1.91			0.75			1.91	0.00	0.75	
1	pcDNA_1278	GGCCGAC	1.5mM	pcDNA	1.38	1.40		0.77	0.50		1.39	0.01	0.63	0.19
1	pcDNA_12917	CCGCCATGCC	1.5mM	pcDNA	3.87			6.09			3.87		6.09	
1	pcDNA_13523	TTGCCTTGTT	1.5mM	pcDNA	1.42	1.40		1.73	1.46		1.41	0.01	1.60	0.19
1	pcDNA_14323	GCTCTAGGGG	1.5mM	pcDNA	21.90	34.60		20.50	17.80		28.25	8.98	19.15	1.91
1	pcDNA_1683	GGGGCAC	1.5mM	pcDNA	5.26	7.92		4.86	5.86		6.59	1.88	5.36	0.71
1	pcDNA_20317	CCAGTCCGCCC	1.5mM	pcDNA	2.83	4.84		1.59	1.32		3.84	1.42	1.46	0.19
1	pcDNA_21088	GGGGGAGGGGC	1.5mM	pcDNA	17.90	29.80		17.60	18.90		23.85	8.41	18.25	0.92
1	pcDNA_22656	CGCCACAACAT	1.5mM	pcDNA	5.50	8.62		4.43	1.66		7.06	2.21	3.05	1.96
1	pcDNA_30638	TGGGGTGGGGCA	1.5mM	pcDNA	42.80	44.50		20.30	16.90		43.65	1.20	18.60	2.40
1	pcDNA_5476	GTGCCAAA	1.5mM	pcDNA	1.37			1.47			1.37	0.00	1.47	
1	pcDNA_6855	AATAATCAA	1.5mM	pcDNA	3.92			2.65			3.92	0.00	2.65	
1	pcDNA_7313	GGAAGGCAC	1.5mM	pcDNA	7.63	8.89		6.31	4.84		8.26	0.89	5.58	1.04

target = Plasmid with exact match for primer

pcDNA.1 = Post-amplification concentration (ng/uL) of DNA from plasmid pcDNA in the 1st experimental replicate. Concentration measued using the Qubit dsDNA HS Assay Kit (Thermo Fisher Scientific)

S1 Table: Primer Amplification Data (continued)

Round	primer name	Primer sequence	Primer molarity	target	pcDNA.1	pcDNA.2	pcDNA.3	pLTR.1	pLTR.2	pLTR.3	Mean Amplification for pcDNA	Standard deviation for pcDNA	Mean Amplification for pLTR	Standard deviation for pLTR
1	pcDNA_8555	GGGGGAGGA	1.5mM	pcDNA	22.30	32.20		44.30	40.40		27.25	7.00	42.35	2.76
1	pLTR_10496	CTAAACGAG	1.5mM	pLTR	1.33	2.00		2.06	2.04		1.67	0.47	2.05	0.01
1	pLTR_12098	GGAAGGAAC	1.5mM	pLTR	12.50	16.10		11.50	8.72		14.30	2.55	10.11	1.97
1	pLTR_14165	GGCTAGAACA	1.5mM	pLTR	18.80	20.90		13.00	10.50		19.85	1.48	11.75	1.77
1	pLTR_1869	TGGTTGG	1.5mM	pLTR	0.99	0.93		1.00	0.84		0.96	0.04	0.92	0.11
1	pLTR_19903	GGGGTCAGATA	1.5mM	pLTR	34.20	44.50		24.70	15.80		39.35	7.28	20.25	6.29
1	pLTR_19941	GGCCCCAATGG	1.5mM	pLTR	12.70	15.40		12.40	15.40		14.05	1.91	13.90	2.12
1	pLTR_27737	GGTGTGGGAGGT	1.5mM	pLTR	47.50	51.00		59.00	55.00		49.25	2.47	57.00	2.83
1	pLTR_29117	CCAGTCCCCCCT	1.5mM	pLTR	1.37			1.61			1.37	0.00	1.61	
1	pLTR_29887	ATTGTTGTTGTT	1.5mM	pLTR	6.57			5.97			6.57	0.00	5.97	
1	pLTR_30742	CTGTGGAAATAA	1.5mM	pLTR	8.38	13.10		14.20	7.19		10.74	3.34	10.70	4.96
1	pLTR_6164	GGTGGAGG	1.5mM	pLTR	10.50	13.80		19.30	25.20		12.15	2.33	22.25	4.17
1	pLTR_695	TGGGGAA	1.5mM	pLTR	3.83			4.61			3.83	0.00	4.61	
1	pLTR_76	ATAGGA	1.5mM	pLTR	1.43			1.47			1.43	0.00	1.47	
1	pLTR_9649	GTTTGACAG	1.5mM	pLTR	2.74			1.22			2.74	0.00	1.22	
1	pcDNA_1278	GGCCGAC	2.5mM	pcDNA	3.07	2.83		1.24	1.04		2.95	0.17	1.14	0.14
1	pcDNA_12917	CCGCCATGCC	2.5mM	pcDNA	7.88			8.53			7.88	0.00	8.53	
1	pcDNA_13523	TTGCCTTGTT	2.5mM	pcDNA	3.54	2.52		4.11	3.09		3.03	0.72	3.60	0.72
1	pcDNA_14323	GCTCTAGGGG	2.5mM	pcDNA	42.20	48.30		29.50	32.30		45.25	4.31	30.90	1.98
1	pcDNA_1683	GGGGCAC	2.5mM	pcDNA	9.90	13.60		10.20	11.80		11.75	2.62	11.00	1.13
1	pcDNA_20317	CCAGTCCGCCC	2.5mM	pcDNA	3.86	2.36		1.88	2.13		3.11	1.06	2.01	0.18
1	pcDNA_21088	GGGGGAGGGGC	2.5mM	pcDNA	38.80	37.30		35.10	31.00		38.05	1.06	33.05	2.90
1	pcDNA_22656	CGCCACAACAT	2.5mM	pcDNA	12.40	9.07		5.75	2.67		10.74	2.35	4.21	2.18
1	pcDNA_30638	TGGGGTGGGGCA	2.5mM	pcDNA	54.00	44.80		28.30	26.80		49.40	6.51	27.55	1.06
1	pcDNA_5476	GTGCCAAA	2.5mM	pcDNA	3.38			2.75			3.38	0.00	2.75	
1	pcDNA_6855	AATAATCAA	2.5mM	pcDNA	6.20			5.98			6.20	0.00	5.98	
1	pcDNA_7313	GGAAGGCAC	2.5mM	pcDNA	15.50	14.50		11.50	12.10		15.00	0.71	11.80	0.42
1	pcDNA_8555	GGGGGAGGA	2.5mM	pcDNA	53.00	51.00		77.10	60.00		52.00	1.41	68.55	12.09
1	pLTR_10496	CTAAACGAG	2.5mM	pLTR	3.28	4.43		4.17	3.98		3.86	0.81	4.08	0.13
1	pLTR_12098	GGAAGGAAC	2.5mM	pLTR	26.30	28.60		20.80	24.70		27.45	1.63	22.75	2.76
1	pLTR_14165	GGCTAGAACA	2.5mM	pLTR	32.20	23.30		22.80	19.20		27.75	6.29	21.00	2.55
1	pLTR_1869	TGGTTGG	2.5mM	pLTR	2.63	1.84		1.73	1.73		2.24	0.56	1.73	0.00
1	pLTR_19903	GGGGTCAGATA	2.5mM	pLTR	56.00	33.80		31.70	25.10		44.90	15.70	28.40	4.67
1	pLTR_19941	GGCCCCAATGG	2.5mM	pLTR	21.20	19.20		19.50	19.50		20.20	1.41	19.50	0.00
1	pLTR_27737	GGTGTGGGAGGT	2.5mM	pLTR	71.80	65.00		80.80	91.20		68.40	4.81	86.00	7.35
1	pLTR_29117	CCAGTCCCCCCT	2.5mM	pLTR	1.68			1.73			1.68	0.00	1.73	
1	pLTR_29887	ATTGTTGTTGTT	2.5mM	pLTR	7.16			9.69			7.16	0.00	9.69	
1	pLTR_30742	CTGTGGAAATAA	2.5mM	pLTR	10.70	6.70		18.70	10.60		8.70	2.83	14.65	5.73
1	pLTR_6164	GGTGGAGG	2.5mM	pLTR	26.20	31.80		44.60	48.90		29.00	3.96	46.75	3.04
1	pLTR_695	TGGGGAA	2.5mM	pLTR	8.27			10.60			8.27	0.00	10.60	
1	pLTR_76	ATAGGA	2.5mM	pLTR	3.04			2.81			3.04	0.00	2.81	

target = Plasmid with exact match for primer
pcDNA.1 = Post-amplification concentration (ng/uL) of DNA from plasmid pcDNA in the 1st experimental replicate. Concentration measued using the Qubit dsDNA HS Assay Kit (Thermo Fisher Scientific)

S1 Table: Primer Amplification Data (continued)

Round	primer name	Primer sequence	Primer molarity	target	pcDNA.1	pcDNA.2	pcDNA.3	pLTR.1	pLTR.2	pLTR.3	Mean Amplification for pcDNA	Standard deviation for pcDNA	Mean Amplification for pLTR	Standard deviation for pLTR
1	pLTR_9649	GTTTGACAG	2.5mM	pLTR	5.92			4.02			5.92	0.00	4.02	
2	pcDNA_47	CGGTAG	0.5mM	pcDNA	0.58	0.55		0.53	0.38		0.57	0.03	0.45	0.11
2	pcDNA_61	GCAAAT	0.5mM	pcDNA	0.20	0.39		0.24	0.26		0.29	0.13	0.25	0.01
2	pcDNA_102	GGCGACC	0.5mM	pcDNA	1.10	0.32		0.29	0.22		0.71	0.55	0.25	0.04
2	pcDNA_151	ACGGGCA	0.5mM	pcDNA	0.46	0.84		0.25	0.38		0.65	0.27	0.32	0.09
2	pcDNA_208	AGGGCGG	0.5mM	pcDNA	2.09	1.98		1.09	1.15		2.04	0.08	1.12	0.04
2	pcDNA_702	TGACGTA	0.5mM	pcDNA	0.18	0.26		0.26	0.64		0.22	0.06	0.45	0.27
2	pcDNA_800	GTTGCCT	0.5mM	pcDNA	0.30	0.35		0.37	0.44		0.33	0.03	0.40	0.05
2	pcDNA_985	TGCCCGT	0.5mM	pcDNA	0.27	0.35		0.32	0.40		0.31	0.06	0.36	0.05
2	pcDNA_1246	CCTTTCC	0.5mM	pcDNA	0.24	0.33		0.50	0.31		0.29	0.06	0.41	0.14
2	pcDNA_1399	TACGTCA	0.5mM	pcDNA	0.32	0.49		0.35	0.28		0.41	0.12	0.31	0.05
2	pcDNA_1500	CCTCATT	0.5mM	pcDNA	0.40	0.36		0.39	0.47		0.38	0.03	0.43	0.06
2	pcDNA_1598	CCGCCCT	0.5mM	pcDNA	0.30	0.33		0.30	0.38		0.31	0.02	0.34	0.06
2	pcDNA_1647	TGCCCGC	0.5mM	pcDNA	0.24	0.36		0.35	0.27		0.30	0.09	0.31	0.06
2	pcDNA_1714	GAGGGCC	0.5mM	pcDNA	0.65	0.40		0.42	0.29		0.52	0.18	0.35	0.09
2	pcDNA_1744	TGCCCGG	0.5mM	pcDNA	0.41	0.39		0.46	0.40		0.40	0.02	0.43	0.05
2	pcDNA_3452	CCCAACTT	0.5mM	pcDNA	0.32	0.25		0.75	0.33		0.29	0.05	0.54	0.29
2	pcDNA_3669	TGGGGTGC	0.5mM	pcDNA	0.94	1.04		1.13	0.74		0.99	0.07	0.93	0.28
2	pcDNA_4640	TTGGGCGG	0.5mM	pcDNA	3.90	3.39		1.86	1.61		3.65	0.36	1.74	0.18
2	pcDNA_4805	GTTGGAGG	0.5mM	pcDNA	1.05	1.18		2.08	1.74		1.12	0.09	1.91	0.24
2	pcDNA_5031	AGTTGGGG	0.5mM	pcDNA	0.86	0.71		0.94	1.25		0.79	0.10	1.09	0.22
2	pcDNA_6018	TCCTTTCC	0.5mM	pcDNA	0.63	0.28		0.40	0.39		0.45	0.25	0.39	0.01
2	pcDNA_6750	CCCTCGCCC	0.5mM	pcDNA	0.30	0.28		0.40	0.17		0.29	0.02	0.28	0.17
2	pcDNA_7776	GTCGTTGGG	0.5mM	pcDNA	4.25	7.49		3.22	3.75		5.87	2.29	3.49	0.37
2	pcDNA_8825	GAGGGCGAC	0.5mM	pcDNA	0.70	0.59		0.55	0.37		0.64	0.08	0.46	0.12
2	pcDNA_13049	TCCTCGCCCT	0.5mM	pcDNA	0.41	0.30		0.28	0.42		0.35	0.08	0.35	0.10
2	pcDNA_15899	CCCACGCGCC	0.5mM	pcDNA	0.82	0.86		0.68	1.05		0.84	0.03	0.87	0.26
2	pcDNA_17714	CCGCCTCTCC	0.5mM	pcDNA	1.25	0.62		0.43	0.31		0.94	0.44	0.37	0.08
2	pcDNA_18454	CTCCTCGCCC	0.5mM	pcDNA	0.53	0.57		0.41	0.46		0.55	0.03	0.43	0.04
2	pcDNA_20474	CGCCTACCGCC	0.5mM	pcDNA	1.06	0.57		0.84	0.57		0.81	0.35	0.70	0.19
2	pcDNA_22844	TCACTGCCCGC	0.5mM	pcDNA	0.64	0.69		1.22	0.88		0.67	0.03	1.05	0.24
2	pcDNA_22955	GCTCCTCGCCC	0.5mM	pcDNA	0.55	0.42		0.59	0.34		0.49	0.09	0.47	0.18
2	pcDNA_23720	CCCAGTCCGCC	0.5mM	pcDNA	0.54	0.56		0.50	0.39		0.55	0.01	0.45	0.08
2	pcDNA_24610	AGCCCTCCCGT	0.5mM	pcDNA	0.80	0.76		0.48	0.49		0.78	0.03	0.49	0.01
2	pcDNA_25156	TGGGAGTTTGTT	0.5mM	pcDNA	1.10	1.38		1.13	0.96		1.24	0.20	1.04	0.12
2	pcDNA_25273	TCGTTGGGCGGT	0.5mM	pcDNA	1.55	2.01		1.07	0.79		1.78	0.33	0.93	0.20
2	pcDNA_26319	GGGGTCTTTGCT	0.5mM	pcDNA	1.44	3.42		0.72	0.84		2.43	1.40	0.78	0.08
2	pcDNA_26389	ACTGTCCTTTCC	0.5mM	pcDNA	0.20	0.49		0.41	0.57		0.35	0.20	0.49	0.11
2	pcDNA_27329	TGCTGGAGTTCG	0.5mM	pcDNA	0.72	0.85		0.61	0.67		0.79	0.09	0.64	0.04
2	pcDNA_27339	GGCAGTACATCT	0.5mM	pcDNA	1.05	0.68		1.87	0.88		0.87	0.26	1.37	0.70
2	pcDNA_27450	AGCCAGGCGGGC	0.5mM	pcDNA	0.91	1.64		0.96	1.30		1.28	0.52	1.13	0.24

target = Plasmid with exact match for primer

pcDNA.1 = Post-amplification concentration (ng/uL) of DNA from plasmid pcDNA in the 1st experimental replicate. Concentration measued using the Qubit dsDNA HS Assay Kit (Thermo Fisher Scientific)

S1 Table: Primer Amplification Data (continued)

Round	primer name	Primer sequence	Primer molarity	target	pcDNA.1	pcDNA.2	pcDNA.3	pLTR.1	pLTR.2	pLTR.3	Mean Amplification for pcDNA	Standard deviation for pcDNA	Mean Amplification for pLTR	Standard deviation for pLTR
2	pcDNA_27824	GGCGAGGGCGAG	0.5mM	pcDNA	24.60	22.80		13.60	15.00		23.70	1.27	14.30	0.99
2	pcDNA_27916	GTGTGTTGGAGG	0.5mM	pcDNA	13.00	16.30		22.90	18.70		14.65	2.33	20.80	2.97
2	pcDNA_28359	TATTCTGGGGGG	0.5mM	pcDNA	1.23	0.97		1.42	0.65		1.10	0.18	1.03	0.55
2	pcDNA_28513	CTCGCCCTCGCC	0.5mM	pcDNA	0.50	0.85		0.75	0.82		0.68	0.25	0.78	0.05
2	pcDNA_28631	GGGTGTCGCCCT	0.5mM	pcDNA	0.81	0.88		0.70	0.74		0.84	0.05	0.72	0.03
2	pcDNA_29636	TGCCAGGCGGGC	0.5mM	pcDNA	0.74	1.07		0.87	0.92		0.91	0.23	0.89	0.04
2	pcDNA_29910	TCGGTCGTTCGG	0.5mM	pcDNA	1.37	1.15		0.91	0.57		1.26	0.16	0.74	0.24
2	pcDNA_30246	GTCGTTGGGCGG	0.5mM	pcDNA	6.80	8.02		3.00	3.53		7.41	0.86	3.27	0.37
2	pLTR_31648	CCCTCA	0.5mM	pLTR	0.15	0.27		0.22	1.03		0.21	0.08	0.63	0.57
2	pLTR_31665	TACTAC	0.5mM	pLTR	0.33	0.39		0.40	0.32		0.36	0.04	0.36	0.06
2	pLTR_31724	GGGGACC	0.5mM	pLTR	0.25	0.38		0.61	0.40		0.32	0.09	0.51	0.15
2	pLTR_31808	ACCCCTT	0.5mM	pLTR	0.15	0.53		0.27	0.46		0.34	0.27	0.36	0.14
2	pLTR_32152	GGGCTGG	0.5mM	pLTR	1.99	1.64		1.06	1.24		1.82	0.25	1.15	0.13
2	pLTR_32279	GGTAGGG	0.5mM	pLTR	1.61	1.45		2.30	2.32		1.53	0.11	2.31	0.01
2	pLTR_32328	AGGCCCC	0.5mM	pLTR	0.35	0.43		0.29	0.29		0.39	0.05	0.29	0.00
2	pLTR_32706	GAGAGGG	0.5mM	pLTR	0.57	0.74		0.86	0.61		0.65	0.13	0.73	0.18
2	pLTR_32714	CACAGAG	0.5mM	pLTR	0.29	0.44		0.41	0.28		0.36	0.11	0.35	0.09
2	pLTR_32931	CCCGGGC	0.5mM	pLTR	0.71	0.64		0.63	0.91		0.67	0.05	0.77	0.20
2	pLTR_33115	GGGGTGA	0.5mM	pLTR	3.21	2.33		3.16	2.00		2.77	0.62	2.58	0.82
2	pLTR_33150	GCCGCCC	0.5mM	pLTR	0.32	0.38		0.36	0.35		0.35	0.05	0.36	0.01
2	pLTR_33527	AAGGGGT	0.5mM	pLTR	0.47	0.32		0.54	0.89		0.39	0.11	0.71	0.25
2	pLTR_35334	ACAGCCCC	0.5mM	pLTR	0.76	0.33		1.04	0.23		0.54	0.31	0.64	0.57
2	pLTR_35959	AAAAACCC	0.5mM	pLTR	0.96	0.24		0.37	0.29		0.60	0.51	0.33	0.06
2	pLTR_36463	GGACCCCC	0.5mM	pLTR	0.40	0.39		0.49	0.47		0.40	0.01	0.48	0.01
2	pLTR_36586	CCAGGGCC	0.5mM	pLTR	1.24	1.23		0.74	0.88		1.24	0.01	0.81	0.10
2	pLTR_37828	GGGGGTCC	0.5mM	pLTR	1.05	1.21		0.90	0.69		1.13	0.11	0.79	0.15
2	pLTR_38270	GGAACCCA	0.5mM	pLTR	0.29	0.21		0.46	0.26		0.25	0.06	0.36	0.14
2	pLTR_38939	GGACCCCCC	0.5mM	pLTR	0.47	0.28		0.45	0.35		0.38	0.13	0.40	0.07
2	pLTR_40559	GGGACTGGG	0.5mM	pLTR	7.36	12.00		9.89	7.58		9.68	3.28	8.74	1.63
2	pLTR_45080	TTCGGTGGGG	0.5mM	pLTR	5.31	5.58		5.57	5.47		5.45	0.19	5.52	0.07
2	pLTR_51071	GACGACCCCC	0.5mM	pLTR	0.45	0.35		0.57	0.31		0.40	0.07	0.44	0.19
2	pLTR_51545	CCAATACGCCC	0.5mM	pLTR	0.42	0.19		0.41	0.39		0.31	0.17	0.40	0.02
2	pLTR_51847	GGGCTGCAGGG	0.5mM	pLTR	7.12	15.30		9.18	15.10		11.21	5.78	12.14	4.19
2	pLTR_52316	CCACAGCCCCC	0.5mM	pLTR	0.52	0.34		0.42	0.29		0.43	0.13	0.35	0.09
2	pLTR_53896	GGGGTTCGGTC	0.5mM	pLTR	1.15	2.20		0.71	0.85		1.68	0.74	0.78	0.10
2	pLTR_54991	ACACCTCCCCC	0.5mM	pLTR	0.50	0.33		0.49	0.37		0.42	0.12	0.43	0.08
2	pLTR_56078	AAATCAGCCCG	0.5mM	pLTR	0.72	0.47		0.58	0.60		0.60	0.17	0.59	0.02
2	pLTR_56359	ACTACACGGCC	0.5mM	pLTR	1.30	2.37		0.82	1.14		1.84	0.76	0.98	0.23
2	pLTR_56732	CCGAACCCCGC	0.5mM	pLTR	0.50	0.68		0.43	0.38		0.59	0.13	0.40	0.04
2	pLTR_58164	ACCGAACCCCG	0.5mM	pLTR	0.49	0.48		0.50	0.60		0.48	0.01	0.55	0.08
2	pLTR_58549	GTTGGATGGAGT	0.5mM	pLTR	0.87	1.07		1.69	1.43		0.97	0.14	1.56	0.18

target = Plasmid with exact match for primer

pcDNA.1 = Post-amplification concentration (ng/uL) of DNA from plasmid pcDNA in the 1st experimental replicate. Concentration measued using the Qubit dsDNA HS Assay Kit (Thermo Fisher Scientific)

S1 Table: Primer Amplification Data (continued)

Round	primer name	Primer sequence	Primer molarity	target	pcDNA.1	pcDNA.2	pcDNA.3	pLTR.1	pLTR.2	pLTR.3	Mean Amplification for pcDNA	Standard deviation for pcDNA	Mean Amplification for pLTR	Standard deviation for pLTR
2	pLTR_58675	GCGGGCGTATTG	0.5mM	pLTR	3.29	5.92		1.18	0.78		4.61	1.86	0.98	0.28
2	pLTR_58977	TGGCCTGGGCGG	0.5mM	pLTR	6.96	5.91		9.34	4.92		6.44	0.74	7.13	3.13
2	pLTR_59052	GCGGGGTTCGGT	0.5mM	pLTR	1.36	2.30		1.43	4.37		1.83	0.66	2.90	2.08
2	pLTR_60579	GGCGGTGGGCGT	0.5mM	pLTR	5.63	7.50		2.01	3.08		6.57	1.32	2.55	0.76
2	pLTR_61207	CCAATACGCCCG	0.5mM	pLTR	0.48	0.48		0.41	0.45		0.48	0.00	0.43	0.03
2	pLTR_61617	GGGGTCTCGGTG	0.5mM	pLTR	5.75	5.72		24.10	3.38		5.74	0.02	13.74	14.65
2	pLTR_61665	AGTCGGGCTTGT	0.5mM	pLTR	0.91	1.75		0.81	1.63		1.33	0.59	1.22	0.58
2	pLTR_62004	AGCTGATTGGGG	0.5mM	pLTR	18.10	23.30		11.70	15.70		20.70	3.68	13.70	2.83
2	pLTR_62908	CTTTGGGCAGGG	0.5mM	pLTR	8.65	12.60		9.33	11.40		10.63	2.79	10.37	1.46
2	pLTR_63257	CTCCGGTGGTGA	0.5mM	pLTR	6.97	7.90		6.16	6.97		7.44	0.66	6.57	0.57
2	pLTR_63897	GGATGGGGGCTG	0.5mM	pLTR	9.88	12.90		10.90	13.80		11.39	2.14	12.35	2.05
2	pLTR_63950	GTAGCTCCGGTG	0.5mM	pLTR	1.69	2.43		2.64	2.17		2.06	0.52	2.41	0.33
2	pLTR_64090	CACGGTGTTGGG	0.5mM	pLTR	11.70	10.30		7.48	5.04		11.00	0.99	6.26	1.73
2	pLTR_64180	ACCACGCCCACC	0.5mM	pLTR	0.46	0.38		0.55	0.39		0.42	0.05	0.47	0.11
2	pLTR_64985	GTTGGGAGTTTC	0.5mM	pLTR	0.36	0.70		0.44	0.91		0.53	0.23	0.67	0.33
3	pcDNA_6732	GGGGAGGGG	0.5mM	pcDNA	5.84	2.90		7.19	6.51		4.37	2.08	6.85	0.48
3	pcDNA_13378	GGGTGGGGCA	0.5mM	pcDNA	9.13	4.83		3.29	3.28		6.98	3.04	3.29	0.01
3	pcDNA_13870	GGGAGGGGCA	0.5mM	pcDNA	8.48	6.85		4.31	5.19		7.67	1.15	4.75	0.62
3	pcDNA_19358	GGTGGGGCAGG	0.5mM	pcDNA	16.00	18.20		15.40	16.60		17.10	1.56	16.00	0.85
3	pcDNA_19877	GGGAGAGGCGG	0.5mM	pcDNA	55.00	57.00		45.60	43.20		56.00	1.41	44.40	1.70
3	pcDNA_20924	GTTGGGGTGGG	0.5mM	pcDNA	34.70	36.50		33.70	44.70		35.60	1.27	39.20	7.78
3	pcDNA_21304	GGGTGGGGCAG	0.5mM	pcDNA	22.70	10.50		15.10	5.57		16.60	8.63	10.34	6.74
3	pcDNA_21546	TGGGGTGGGCG	0.5mM	pcDNA	25.40	16.90		12.40	5.89		21.15	6.01	9.15	4.60
3	pcDNA_22525	GGCGCGTGGGG	0.5mM	pcDNA	41.20	29.10		37.00	20.60		35.15	8.56	28.80	11.60
3	pcDNA_22551	GGGCGAGGGCG	0.5mM	pcDNA	65.00	55.00		35.80	23.30		60.00	7.07	29.55	8.84
3	pcDNA_22840	GGGGAGAGGCG	0.5mM	pcDNA	50.00	44.50		41.40	31.60		47.25	3.89	36.50	6.93
3	pcDNA_22971	GCGGGGAGAGG	0.5mM	pcDNA	15.90	12.60		20.80	8.51		14.25	2.33	14.66	8.69
3	pcDNA_24957	GGGGTGGGGCA	0.5mM	pcDNA	14.10	10.60		5.58	6.13		12.35	2.47	5.86	0.39
3	pcDNA_25228	ATGTGGTCGGGG	0.5mM	pcDNA	25.30	32.00		17.70	17.90		28.65	4.74	17.80	0.14
3	pcDNA_25350	GCGAGGGCGAGG	0.5mM	pcDNA	39.00	35.30		26.30	27.70		37.15	2.62	27.00	0.99
3	pcDNA_25366	GGGAGGGGCAAA	0.5mM	pcDNA	12.80	13.50		15.40	21.70		13.15	0.49	18.55	4.45
3	pcDNA_26085	GGGAGAGGCGGT	0.5mM	pcDNA	29.50	25.70		18.20	22.70		27.60	2.69	20.45	3.18
3	pcDNA_26209	GGGCGGACTGGG	0.5mM	pcDNA	41.80	51.00		31.80	42.10		46.40	6.51	36.95	7.28
3	pcDNA_26275	GGATGGAGGCGG	0.5mM	pcDNA	47.50	37.70		38.50	18.10		42.60	6.93	28.30	14.42
3	pcDNA_26354	GGGGATGCGGTG	0.5mM	pcDNA	63.10	42.90		39.20	26.90		53.00	14.28	33.05	8.70
3	pcDNA_26599	GGTGGGCCAGGG	0.5mM	pcDNA	31.40	28.60		29.30	19.20		30.00	1.98	24.25	7.14
3	pcDNA_26808	AGGGGATCGGGA	0.5mM	pcDNA	43.70	35.90		40.40	27.80		39.80	5.52	34.10	8.91
3	pcDNA_27205	AGGGCGCGTGGG	0.5mM	pcDNA	49.90	55.00		58.00	40.90		52.45	3.61	49.45	12.09
3	pcDNA_27206	GCGCGGGGAGAG	0.5mM	pcDNA	27.80	12.90		38.30	11.40		20.35	10.54	24.85	19.02
3	pcDNA_27357	TGGATGGAGGCG	0.5mM	pcDNA	19.90	23.90		14.80	13.10		21.90	2.83	13.95	1.20
3	pcDNA_27509	GCTGGGGATGCG	0.5mM	pcDNA	16.80	17.10		6.92	4.10		16.95	0.21	5.51	1.99

target = Plasmid with exact match for primer

pcDNA.1 = Post-amplification concentration (ng/uL) of DNA from plasmid pcDNA in the 1st experimental replicate. Concentration measued using the Qubit dsDNA HS Assay Kit (Thermo Fisher Scientific)

S1 Table: Primer Amplification Data (continued)

Round	primer name	Primer sequence	Primer molarity	target	pcDNA.1	pcDNA.2	pcDNA.3	pLTR.1	pLTR.2	pLTR.3	Mean Amplification for pcDNA	Standard deviation for pcDNA	Mean Amplification for pLTR	Standard deviation for pLTR
3	pcDNA_27738	CAATGGGCGGGG	0.5mM	pcDNA	31.70	40.30		24.50	23.70		36.00	6.08	24.10	0.57
3	pcDNA_27776	GGGGTGGGCGAA	0.5mM	pcDNA	35.60	34.60		21.00	27.70		35.10	0.71	24.35	4.74
3	pcDNA_28042	GGGTGGGCCAGG	0.5mM	pcDNA	13.90	16.60		12.50	14.60		15.25	1.91	13.55	1.48
3	pcDNA_28153	GTACGGTGGGAG	0.5mM	pcDNA	22.50	28.90		24.50	29.60		25.70	4.53	27.05	3.61
3	pcDNA_28208	GAAGGCACGGGG	0.5mM	pcDNA	28.40	20.70		22.70	10.60		24.55	5.44	16.65	8.56
3	pcDNA_28226	ATGGGGTGGAGA	0.5mM	pcDNA	59.00	40.50		92.90	46.00		49.75	13.08	69.45	33.16
3	pcDNA_28269	GTGGGAGTGGCA	0.5mM	pcDNA	42.10	36.00		31.20	16.80		39.05	4.31	24.00	10.18
3	pcDNA_28357	AGGGCGAGGGCG	0.5mM	pcDNA	46.30	55.00		30.70	28.50		50.65	6.15	29.60	1.56
3	pcDNA_28401	AATGGGGCGGAG	0.5mM	pcDNA	40.90	46.00		55.00	45.20		43.45	3.61	50.10	6.93
3	pcDNA_28633	GGGAGGATTGGG	0.5mM	pcDNA	57.30	51.00		51.00	32.30		54.15	4.45	41.65	13.22
3	pcDNA_28958	GGGGTCGTTGGG	0.5mM	pcDNA	32.30	32.10		25.70	15.10		32.20	0.14	20.40	7.50
3	pcDNA_29069	GTCGGGGTAGCG	0.5mM	pcDNA	5.25	4.93		3.13	2.11		5.09	0.23	2.62	0.72
3	pcDNA_29217	GGAAGGGCCGAG	0.5mM	pcDNA	21.80	17.80		12.60	10.80		19.80	2.83	11.70	1.27
3	pcDNA_29486	CGCGCGGGGAGA	0.5mM	pcDNA	18.40	11.30		13.20	16.80		14.85	5.02	15.00	2.55
3	pcDNA_29582	GGATGCGGTGGG	0.5mM	pcDNA	27.90	45.50		29.70	30.20		36.70	12.45	29.95	0.35
3	pcDNA_29659	GCGGGGAGAGGC	0.5mM	pcDNA	20.80	19.20		17.90	21.10		20.00	1.13	19.50	2.26
3	pcDNA_30367	GGTGAGCGTGGG	0.5mM	pcDNA	56.60	41.80		68.70	25.80		49.20	10.47	47.25	30.33
3	pcDNA_30390	GGGCGGTAGGCG	0.5mM	pcDNA	29.00	20.80		13.10	7.44		24.90	5.80	10.27	4.00
3	pcDNA_30447	GGGGATCGGGAG	0.5mM	pcDNA	25.40	17.60		25.20	13.30		21.50	5.52	19.25	8.41
3	pcDNA_30584	GGCGCGTGGGGA	0.5mM	pcDNA	67.00	78.30		76.80	62.10		72.65	7.99	69.45	10.39
3	pcDNA_31433	AGTTGGGGTGGG	0.5mM	pcDNA	48.70	52.60		67.30	51.00		50.65	2.76	59.15	11.53
3	pcDNA_31528	GGGGCGGAGTTG	0.5mM	pcDNA	23.80	16.00		13.70	6.57		19.90	5.52	10.14	5.04
3	pLTR_47269	GGGGAGGAGG	0.5mM	pLTR	23.80	15.20		27.30	28.90		19.50	6.08	28.10	1.13
3	pLTR_47895	GGGGTAGGGG	0.5mM	pLTR	23.80	17.30		19.20	17.50		20.55	4.60	18.35	1.20
3	pLTR_48127	GGGAGGAGGG	0.5mM	pLTR	35.10	28.60		38.80	39.60		31.85	4.60	39.20	0.57
3	pLTR_50519	GGGGTGGGGA	0.5mM	pLTR	15.80	12.40		18.50	18.50		14.10	2.40	18.50	0.00
3	pLTR_52636	GGGGCGGCAGG	0.5mM	pLTR	41.50	37.30		23.50	28.80		39.40	2.97	26.15	3.75
3	pLTR_53226	AGGGGTAGGGG	0.5mM	pLTR	36.00	30.20		33.10	33.10		33.10	4.10	33.10	0.00
3	pLTR_53662	GAGGGAGTGGG	0.5mM	pLTR	51.00	33.80		57.00	28.80		42.40	12.16	42.90	19.94
3	pLTR_54872	GGTGTGGGAGG	0.5mM	pLTR	42.20	28.70		48.20	33.40		35.45	9.55	40.80	10.47
3	pLTR_56476	GAGGAGGGAGG	0.5mM	pLTR	32.60	20.00		41.40	24.70		26.30	8.91	33.05	11.81
3	pLTR_56509	GGGGAGTGGCG	0.5mM	pLTR	53.00	53.00		44.90	46.00		53.00	0.00	45.45	0.78
3	pLTR_56594	GGAGGTGTGGG	0.5mM	pLTR	58.00	51.00		55.80	31.00		54.50	4.95	43.40	17.54
3	pLTR_56787	GGGGAGGTGTG	0.5mM	pLTR	20.70	12.60		21.90	11.40		16.65	5.73	16.65	7.42
3	pLTR_58475	GAGGTGTGGGAG	0.5mM	pLTR	31.20	27.10		43.90	45.20		29.15	2.90	44.55	0.92
3	pLTR_58585	AGGTGTGGGAGG	0.5mM	pLTR	33.70	39.60		56.00	46.70		36.65	4.17	51.35	6.58
3	pLTR_58664	GGCGGGACTGGG	0.5mM	pLTR	41.10	32.80		29.00	39.20		36.95	5.87	34.10	7.21
3	pLTR_58719	GGGACTGGGGAG	0.5mM	pLTR	30.90	40.10		29.70	40.90		35.50	6.51	35.30	7.92
3	pLTR_58962	GTGGGGTGGGGA	0.5mM	pLTR	39.60	36.10		37.10	52.00		37.85	2.47	44.55	10.54
3	pLTR_59017	GAGGGGACTGGA	0.5mM	pLTR	23.40	25.40		26.40	35.60		24.40	1.41	31.00	6.51
3	pLTR_59047	GGGGAGGTGTGG	0.5mM	pLTR	70.20	52.00		71.00	40.40		61.10	12.87	55.70	21.64

target = Plasmid with exact match for primer

pcDNA.1 = Post-amplification concentration (ng/uL) of DNA from plasmid pcDNA in the 1st experimental replicate. Concentration measued using the Qubit dsDNA HS Assay Kit (Thermo Fisher Scientific)

S1 Table: Primer Amplification Data (continued)

Round	primer name	Primer sequence	Primer molarity	target	pcDNA.1	pcDNA.2	pcDNA.3	pLTR.1	pLTR.2	pLTR.3	Mean Amplification for pcDNA	Standard deviation for pcDNA	Mean Amplification for pLTR	Standard deviation for pLTR
3	pLTR_59170	GAGGGAGTGGGT	0.5mM	pLTR	31.70	27.00		34.90	18.20		29.35	3.32	26.55	11.81
3	pLTR_59590	GGGTGAGTAGGG	0.5mM	pLTR	86.20	72.00		94.90	80.00		79.10	10.04	87.45	10.54
3	pLTR_59609	GGTGGGGAAAAG	0.5mM	pLTR	28.50	31.00		24.80	21.00		29.75	1.77	22.90	2.69
3	pLTR_59716	GGTGAGTAGGGG	0.5mM	pLTR	37.90	11.80		34.40	9.98		24.85	18.46	22.19	17.27
3	pLTR_59817	TCGGGGAGGAGG	0.5mM	pLTR	35.60	24.30		44.90	28.80		29.95	7.99	36.85	11.38
3	pLTR_60002	AGGGGTAGGGGT	0.5mM	pLTR	13.50	12.60		12.90	14.40		13.05	0.64	13.65	1.06
3	pLTR_60226	GGGGAGAAGGAA	0.5mM	pLTR	42.90	29.60		34.50	29.50		36.25	9.40	32.00	3.54
3	pLTR_60467	TGGGTGACGGAG	0.5mM	pLTR	38.00	51.00		35.60	40.60		44.50	9.19	38.10	3.54
3	pLTR_60692	AGAGGGGAGGTA	0.5mM	pLTR	10.00	11.90		14.20	16.90		10.95	1.34	15.55	1.91
3	pLTR_60777	AGGGAGGCGTGG	0.5mM	pLTR	43.90	71.60		45.90	78.00		57.75	19.59	61.95	22.70
3	pLTR_60812	GTCGGGGAGGAG	0.5mM	pLTR	58.00	73.50		77.90	78.30		65.75	10.96	78.10	0.28
3	pLTR_61318	GGGAGGAGGGAG	0.5mM	pLTR	37.30	27.60		55.00	28.10		32.45	6.86	41.55	19.02
3	pLTR_61590	GGAGGGGACTGG	0.5mM	pLTR	30.70	23.20		43.10	20.60		26.95	5.30	31.85	15.91
3	pLTR_61668	TGGCGGTGGGCG	0.5mM	pLTR	40.70	45.60		19.40	12.40		43.15	3.46	15.90	4.95
3	pLTR_61827	GGTGGGGTATCG	0.5mM	pLTR	7.67	4.94		5.05	2.59		6.31	1.93	3.82	1.74
3	pLTR_62009	TGGGGTGGGGAA	0.5mM	pLTR	15.40	20.50		26.10	17.80		17.95	3.61	21.95	5.87
3	pLTR_62178	GGGGCGGCAGGC	0.5mM	pLTR	19.40	19.40		17.30	9.71		19.40	0.00	13.51	5.37
3	pLTR_62577	AGAGGGAGTGGG	0.5mM	pLTR	34.70	26.40		30.80	32.50		30.55	5.87	31.65	1.20
3	pLTR_62928	GGCCTGGGCGGG	0.5mM	pLTR	11.80	7.95		8.49	8.64		9.88	2.72	8.57	0.11
3	pLTR_63038	CGGGGCGGCAGG	0.5mM	pLTR	29.70	25.60		19.20	19.90		27.65	2.90	19.55	0.49
3	pLTR_63109	GGGAGTGGCGAG	0.5mM	pLTR	41.60	33.50		33.80	41.00		37.55	5.73	37.40	5.09
3	pLTR_63515	GGATCGGAGGGG	0.5mM	pLTR	13.60	18.60		17.40	26.80		16.10	3.54	22.10	6.65
3	pLTR_63883	GTCGGGGCGGCA	0.5mM	pLTR	18.30	18.70		11.20	12.90		18.50	0.28	12.05	1.20
3	pLTR_63991	AGAGGGGTAGGG	0.5mM	pLTR	24.30	18.20		30.00	15.10		21.25	4.31	22.55	10.54
3	pLTR_64095	GCGGGACTGGGG	0.5mM	pLTR	30.40	20.80		29.00	14.80		25.60	6.79	21.90	10.04
3	pLTR_64172	GGAAGAGGTGGG	0.5mM	pLTR	29.10	23.20		42.40	22.10		26.15	4.17	32.25	14.35
3	pLTR_64428	GGGGTAGGGGTA	0.5mM	pLTR	9.82	7.85		9.16	5.65		8.84	1.39	7.41	2.48
3	pLTR_64650	GGGGCTGTGGCA	0.5mM	pLTR	16.40	13.40		11.50	7.03		14.90	2.12	9.27	3.16
3	pLTR_64865	TGGGGAGTGGCG	0.5mM	pLTR	29.90	35.50		40.10	20.20		32.70	3.96	30.15	14.07

target = Plasmid with exact match for primer

pcDNA.1 = Post-amplification concentration (ng/uL) of DNA from plasmid pcDNA in the 1st experimental replicate. Concentration measued using the Qubit dsDNA HS Assay Kit (Thermo Fisher Scientific)
