# Supplementary material for: A fast machine-learning-guided primer design pipeline for selective whole genome amplification
Source: PLoS Comput Biol. 2023 Apr 17;19(4):e1010137. doi: 10.1371/journal.pcbi.1010137 (PMC10138271; doi:10.1371/journal.pcbi.1010137)
Supplement: S2 Table — (PDF) [file pcbi.1010137.s003.pdf]

S2 Table: Primer set statistics and sequences

Name	fg/bg ratio	Size	fg gini	fg max distance	fg mean distance	fg std deviation	bg mean distance	Primers
Prev01	0.0538	2	0.600	6.53E+04	7.39E+03	9.24E+03	1.37E+05	AACGAT*A*G, ACGATA*G*A
Prev02	0.0494	3	0.566	4.22E+04	5.03E+03	5.95E+03	1.02E+05	AACGAT*A*G, ACGATA*G*A, TGAGCG*T*A
Prev03	0.0916	4	0.533	3.15E+04	4.10E+03	4.35E+03	4.48E+04	ATCGGT*A*A, TACGAA*G*A, TCATCG*G*T, TATCGG*T*G
Prev04	0.0664	9	0.623	3.33E+04	1.98E+03	2.64E+03	2.99E+04	ACGATA*G*C, CGAAGA*T*A, CACGAT*A*G, ACGATA*G*T, AATTCATAA*T*T, TGAGCG*T*A, GAGCGT*A*A, GCGTTA*G*T, GGCGTT*A*G
Prev05	0.0804	6	0.638	3.77E+04	2.76E+03	3.79E+03	3.43E+04	ACGATA*G*C, CGAAGA*T*A, CACGAT*A*G, ACGATA*G*T, AACGAT*A*G, ACGATA*G*A
Prev06	0.0980	3	0.552	3.13E+04	4.98E+03	5.46E+03	5.08E+04	CGAAC*G*A, CGAAGA*T*A, GCGAA*C*G
