# Supplementary material for: A fast machine-learning-guided primer design pipeline for selective whole genome amplification
Source: PLoS Comput Biol. 2023 Apr 17;19(4):e1010137. doi: 10.1371/journal.pcbi.1010137 (PMC10138271; doi:10.1371/journal.pcbi.1010137)
Supplement: S3 Table — (PDF) [file pcbi.1010137.s004.pdf]

S3 Table: Percent of reads mapping to *Prevotella* and Human (background) sequences (15 Mbp sequenced).

	Reads Mapping to *Prevotella*	Reads Mapping to Background
Control	0.60%	99.12%
Prev01	1.23%	98.70%
Prev02	1.24%	98.70%
Prev03	57.34%	42.36%
Prev04	7.70%	92.05%
Prev05	1.20%	98.74%
Prev06	72.22%	26.02%
