# Supplementary material for: A fast machine-learning-guided primer design pipeline for selective whole genome amplification
Source: PLoS Comput Biol. 2023 Apr 17;19(4):e1010137. doi: 10.1371/journal.pcbi.1010137 (PMC10138271; doi:10.1371/journal.pcbi.1010137)
Supplement: S4 Table — (PDF) [file pcbi.1010137.s005.pdf]

S4 Table: Proportion of called bases matching the *Prevotella* reference genome demonstrates that SWGA does not introduce sequencing errors.

Sample	Bases called	% matching reference
Control	2,430,966	99.9933%
Prev03A	2,932,347	99.9976%
Prev03B	2,987,130	99.9987%
Prev06A	3,048,879	99.9989%
Prev06B	3,119,187	99.9996%
Prev09A	1,642,904	99.9926%
Prev09B	2,635,407	99.9953%
