# Supplementary material for: A fast machine-learning-guided primer design pipeline for selective whole genome amplification
Source: PLoS Comput Biol. 2023 Apr 17;19(4):e1010137. doi: 10.1371/journal.pcbi.1010137 (PMC10138271; doi:10.1371/journal.pcbi.1010137)
Supplement: Attachment — Submitted filename: ReviewerComments.pdf [file pcbi.1010137.s006.pdf]

Response to reviewers

We thank the editors and reviewers for their detailed and constructive assessment of our manuscript. A primary concern from the associate editor and one reviewer was that the model description and some terminology throughout the manuscript were not completely clear. We agree that a clearer and more precise description can only improve the impact of the work and have addressed these comments. The associate editor and both reviewers also found typos that we have now corrected. We are very grateful for all of the useful and constructive comments. There were many other more specific, constructive comments that we have now addressed. To distinguish our responses from the original reviewer comments, we have labeled reviewer comments in **blue** and our responses in **black**.

Reviewer 1: In this manuscript the authors introduce version 2 of the SWGA pipeline which optimizes primer design for selective whole genome amplification. This is a very valuable tool for enriching target DNA for sequencing in cases where samples would typically be dominated by background or host DNA. In the new version, the authors have added several speed ups to the original algorithm and used a machine learning approach to identify efficient primers features. The new additions to the pipeline are substantial and warrant a second paper to introduce these enhancements. The authors demonstrate the performance of swga2.0 by designing and testing 6 primer sets for amplifying Prevotella melaninogenica within a human background. The manuscript is well organized and clearly written in a way that is understandable to a non-computational audience and the best practices section at the end is very helpful.

> **Thank you. We hope it is useful to the community.**

The authors provide publicly available, version-controlled, and well documented code with appropriate documentation on installation and workflow. A local copy of the codebase can easily be installed using pip however it would be assessable to a wider audience if it could be installed using Conda (or similar) with all dependencies. The name of the software is different than what is reported in the paper which may be confusing. There does not appear to be any unit tests.

> **We thank the reviewer for their suggestion to make `swga2.0` more impactful. We have created a conda package, which can be found at `https://anaconda.org/janedwivedi/swga2` in addition to the github repository (`https://github.com/songlab-cal/swga2`). We have also added several sample files that users can download and use to verify that each stage of the program is working properly.**

The sequencing and raw data was not provided with the manuscript and would be required to reproduce the results. The primer sequences were also not provided as far as I can tell.

> **We agree with the reviewer that all of the data presented in this manuscript must be available. We have added the primer set statistics and the data from the plasmid**

amplification experiments to the supplement (Tables S1 and S2 (Table 2)). The sequencing data has been uploaded to Dryad (`https://doi.org/10.5061/dryad.3n5tb2rm2`). These data and tables are now referenced in the results section.

There are not many other tools to directly compare to, so the authors mostly refer to previous results of swga1.0 but they did not do a direct comparison. Because the focus of this paper is on improvements from version 1.0, it would be helpful if the authors provide a summary table that compares the features between the old and new versions.

Thank you for this idea. As suggested, we have now included a table enumerating the differences in features between v1.0 and v2.0 in Table 1 in the revised manuscript (see Table 1 below).

To drive home the benefit of the newly added primer efficacy filter, it would be good to build sets using swga1.0 and evaluate how often the "best" sets contain primers that are predicted to be low efficiency and at what proportion. This would lend support the claims in the paper and would be fairly quick to run without requiring any additional sequencing.

We agree and have added several comparisons between swga1.0 and 2.0 in the revised manuscript. We now demonstrate the impact of the primer efficacy filter in Table 5. The initial stage of both pipelines identifies the same candidate primers, although swga2.0 is more efficient. The primer efficacy filter eliminates over 80% of the primers that would have been used in the swga1.0 pipeline to build primer sets (of the 76 primers that would have been used to build primer sets with swga1.0, only 11 pass the primer efficacy filter). Further, all of ten top-rated primer sets designed by `swga1.0` are made exclusively of primers with low-amplification potential. Thus, all of the top sets designed by swga1.0 contain predicted low-amplification primers.

Furthermore, given that the multiprocessing speedups are another focus of the paper, I recommend that a more rigorous comparison is performed between the two versions. A time was provided for a single run of v2.0 and the time for v1.0 was given as over two weeks. The number of processors and the memory requirements were not provided. It would be good to know how many processors were used and if there are diminishing returns. Was swga1.0 able to find a result after running for 2 weeks or did it get stuck? I believe that I have been able to design SWGA primers in less than two weeks using swga1.0, so it is unclear why it took so long. Perhaps it would be good to try a less complicated design to compare the efficiency between the two versions so that we can better evaluate the improvement of each of the speedups. It would be great to see a breakdown of the estimated time saved due to each of the improvements listed in the section titled: "Computational costs are reduced from weeks to minutes"

Table 1: Differences between `swga1.0` and `swga2.0`.

	Feature	`swga1.0`	`swga2.0`
**(All stages)**	Time to complete (minutes)*	1176	93
	Multiprocessing		✓
	Incorporates forward strand	✓	✓
	Incorporates reverse strand		✓
**(Stage 1)**	Time to complete (minutes)*	100	20
	Process k-mers that exact-match the target	✓	✓
	Process all k-mers in the target		✓
**(Stage 2)**	Time to complete (minutes)*	0.1	37.5
	Filter by melting temp	✓	✓
	Filter by self-dimerization	✓	✓
	Filter by target binding count	✓	✓
	Filter by background binding count	✓	✓
	Filter by target Gini index	✓	✓
	Filter by target to background freq. binding ratio	✓	✓
**(Stage 3)**	Time to complete (minutes)*	NA	5
	Filter by predicted amplification using a trained model		✓
**(Stage 4)**	Time to complete (minutes)*	1076	31
	Filters based on heterodimer formation	✓	✓
	Score sets using heuristic function	✓	
	Score sets using fitted regression model		✓
	Uses min distance between binding sites in the background	✓	
	Uses max distance between binding sites on the target	✓	
	Uses min ratio (target:background) of mean binding site distances		✓
	Uses max ratio (target:background) of binding site frequency		✓
	Uses min ratio (target:background) of coverage approximation		✓
	Uses min mean Gini index of target binding		✓
	Uses max mean Gini index of background binding		✓
	Branch-and-bound search based on heterodimer cliques	✓	✓
	Branch-and-bound based on predict set score		✓

*Primer sets designed to selectively amplify *P. melaninogenica* (*H. sapiens* background) were built on a MacBookPro14.1 (2.5GHz Dual-Core Intel Core i7; Memory 16GB 2133MHz LPDDR3).

This was an omission of important information. We have now run both v1 and v2 on the same computer to directly compare the computational time saved. It is difficult to make perfect comparisons due to the differences in parameters. Thus, we used the simplest (*i.e.* most time efficient) settings for swga1.0 in order to design primer sets for *Prevotella*. We compared this to the time it took to run swga2.0 using the settings we actually used to find the primer sets used in this manuscript. We have broken this down by stage in Table 1. Overall, finding primer sets using swga2.0 is nearly 10 times faster than v1. More importantly, all of the primer sets identified by swga1.0 are dominated by low-amplification primers, regardless of the settings used, such that these sets are likely to perform poorly on the bench.

About half of the primer sets failed using swga2.0. There was some mention in the discussion about a relationship between the successful sets and lower mean binding distance in the target genome, but it would be nice to have a table of all the observed stats for the sets in addition to the parameter settings.

We apologize that this was insufficiently described in the original manuscript. Prior studies have shown that mean binding distance in the target was generally correlated with amplification efficiency. The primer sets designed to amplify *Prevotella* generally follow this trend. However, all of the sets have similarly low mean binding distances so most of the observed variation in amplification success is likely due to yet-to-be-discovered factors. We have made these points more clear in the manuscript. We have also included a description of the primer sets in Table S2 (and see Table 2 below)

Table 2: Primer set stats and sequences

Name	fg/bg ratio	Size	fg gini	fg max distance	fg mean distance	fg std deviation	bg mean distance	Primers
Prev01	0.0538	2	0.600	6.53E+04	7.39E+03	9.24E+03	1.37E+05	AACGAT*A*G, ACGATA*G*A
Prev02	0.0494	3	0.566	4.22E+04	5.03E+03	5.95E+03	1.02E+05	AACGAT*A*G, ACGATA*G*A, TGAGCG*T*A
Prev03	0.0916	4	0.533	3.15E+04	4.10E+03	4.35E+03	4.48E+04	ATCGGT*A*A, TACGAA*G*A, TCATCG*G*T, TATCGG*T*G
Prev04	0.0664	9	0.623	3.33E+04	1.98E+03	2.64E+03	2.99E+04	ACGATA*G*C, CGAAGA*T*A, CACGAT*A*G, ACGATA*G*T, AATTCATAA*T*T, TGAGCG*T*A, GAGCGT*A*A, GCGTTA*G*T, GGCGTT*A*G
Prev05	0.0804	6	0.638	3.77E+04	2.76E+03	3.79E+03	3.43E+04	ACGATA*G*C, CGAAGA*T*A, CACGAT*A*G, ACGATA*G*T, AACGAT*A*G, ACGATA*G*A
Prev06	0.0980	3	0.552	3.13E+04	4.98E+03	5.46E+03	5.08E+04	CGAAC*G*A, CGAAGA*T*A, GCGAA*C*G

The discussion mentions that this approach would work on metagenomic samples (last paragraph). Has this been done and how would swga2.0 be able to design primer sets for such complex backgrounds? How many background genomes could reasonably be added? How would you handle all the potential plasmids in such a sample? The last paragraph of the discussion abruptly introduces a number of new ideas that are difficult to follow. These should be expanded on to provide clarity if they are important or eliminated.

We agree that this was speculative. We have removed this paragraph as we do not have the empirical data to support it.

The EquiPhi29 enzyme is more thermally stable and produces higher yield than Phi29. Using this enzyme may allow longer primers with higher Tm. Could the authors provide some discussion about whether swga2.0 can design appropriate primer sets for use with this enzyme and some recommended parameter adjustments.

This is a good question and we have now added a discussion of this topic. It is likely that most of the parameters in the random forest primer efficacy model will work for both phi29 and other polymerases (EquiPhi29 or Bst). Most of the parameters assess DNA-DNA binding potential and thus should be independent of the polymerase used. Other features are likely to be affected by the polymerase chosen, such as melting temperature. Conservatively, it would be prudent to empirically validate the primer efficacy model using EquiPhi29 or Bst.

Mispriming in the rolling circle plasmid amplification experiment was mentioned but there were no results, and it was unclear if that was incorporated into the model.

We have now included all of the data from the rolling circle plasmid amplification experiments as supplemental Table S1. Table S1 shows the amount of amplification from the target (1 primer binding site) and off-target (no primer binding sites, all amplification is from mispriming) for each primer. Both the on and off-target amplification data were used to build the random forest model.

It would be good to have the number of reads that aligned to the background genome.

This is a good idea. The proportion of reads aligning to the background genome are now presented in Table S3 (see Table 3 below).

Table 3: Percent of reads mapping to Human and Prevotella genomes after 15 Mbp sequenced.

	Reads Mapping to *Prevotella*	Reads Mapping to Background
Control	0.60%	99.12%
Prev01	1.23%	98.70%
Prev02	1.24%	98.70%
Prev03	57.34%	42.36%
Prev04	7.70%	92.05%
Prev05	1.20%	98.74%
Prev06	72.22%	26.02%

The methods do not describe exactly how the target amplification efficiency was calculated.

We apologize that this was not clear. Primer amplification efficiency was predicted using a random forest regression model, which is available for download from our

github (`https://github.com/songlab-cal/swga2`). **We have changed the language throughout the manuscript to clarify that primer amplification efficiency was predicted from an empirically validated model.**

Minor Comments:

Line 10 Abstract: Change "Evaluate" to "evaluates"

This has been fixed.

Page 3 line 5 and 6: Change "ie" to "i.e.,"

This has been fixed.

Page 3 second paragraph: change "as well as identifying" to "and identified"

This has been fixed.

Page 3, 4th line of last paragraph in introduction beginning with "First, active learning" this sentence should be reworded. Something like "First, active machine learning was used to identify features that predict primer and primer set efficiency"

This has been fixed.

Figure 3 caption, last line: change "axis" to "x-axis"

This has been fixed.

Page 8 Section "Empirical evaluation of primer sets": Can you clarify that the FspE1 digestion was to suppress mitochondrial amplification and note whether or not swga2.0 has the option to omit mitochondrial sequences?

This is a good question that is a bit beyond the scope of this manuscript. Nevertheless, it is worth noting as several users are likely to have a similar question. We have now included a brief consideration of both mitochondrial amplification issues and the use of restriction digests. Briefly, primers that bind to mitochondrial sequences are not excluded in swga2.0 as the new program considers both exact match sites and inexact matches that are nevertheless expected to promote amplification. Exact matches can be removed manually as the candidate primers are output to a .csv file at each stage. DNA digestion was not performed in order to suppress mitochondrial amplification

specifically as this was not an issue in this system. Some prior protocols have digested the genomic DNA prior to amplification with some success, while others found no advantage. The idea of selective digestion is to reduce the length of background DNA which gives the target DNA an amplification advantage with phi29 (the length of template correlates with the amount of exponential amplification with phi29). However, we did try to amplify Prevotella using random heptamers from digested samples and gained only a minor target amplification advantage. These data suggest that digestion may not have been necessary in this system, although we did not assess this extensively.

Page 9 first line: Missing reference and version for samtools.

We have added the version number and reference.

Page 9, second paragraph, last line: Change "data from the rounds" to "data from rounds".

This has been fixed.

The text references Figure 3A but there is only a single for Figure 3.

Changed Figure 3A to Figure 3.

The variables described in Stage 4 of the methods are not described until much later in the paper. I would move them up to where they are first mentioned.

Thank you for the suggestion. We have added the following sentence: "The features for this regression are described in Table 4 and explained further in the section Primer set search and scoring function."

Page 13, line 8: Change "eliminate" to "eliminating"

This has been fixed.

Page 14: Sentence "Highthroughput sequencing of the amplification products from the three effective P. melaninogenica primer sets reached 1× coverage across 25–64% the target genome with 50Mbp sequencing effort while only 27% of the M. tuberculosis was covered at 1× at similar sequencing effort after amplification with the most effective primer set (Figure 5)" is confusing. Figure 5 does not contain data about M. tuberculosis so I would move the figure reference closer to the actual result and clarify again that you are referring to M. tuberculosis results from the previous version of swga.

We agree that the writing in the first submission was confusing. We have changed this sentence to read "Highthroughput sequencing of the amplification products from the three effective *P. melaninogenica* primer sets reached 1× coverage across 25–64% the target genome with 50Mbp sequencing effort (Figure 5). In a prior study, the most effective primer set designed by `swga1.0` reach 1× coverage across only 27% of the M. tuberculosis with 50Mbp sequencing effort."

Pg 17 line 9: Change "utilizing using" with "using"

This has been fixed.

Reviewer 2: Yu et al. describe swga2.0, a pipeline that improves upon the original swga program described by Clarke et al. to identify, screen, and choose primers for use in selective whole-genome amplification. The authors apply swga2.0 to identify candidate sWGA primer sets for enrichment of P. melaninogenica and then test the best performing primer sets experimentally.

The manuscript is well-written and easy to follow. Major advances of the new pipeline are greatly reduced computational time (the prior version required weeks on a high-powered computing cluster, the current version purports to requires hours on a personal laptop) and use of machine-learning methods to select primers that are likely to perform well. The model used to predict amplification efficiency (or more accurately, probability of poor amplification) could be very useful for a variety of applications, though experimental data for enzymes other than phi29 would be required. Several companies offer services for highly multiplexed primer design but do not make their code publicly available. The approaches described here could be useful not only for sWGA primer design but for other applications as well, including design of highly multiplexed amplicon panels.

We appreciate your efforts and hope that this project will make SWGA accessible to more researchers.

The manuscript could be improved in several ways as outlined below – namely application to more than one pathogen and explicit comparison of swga2.0 to the original swga program.

We agree with these comments and have added two tables to more directly compare swga1.0 and 2.0. Table 1 (Table 1 below) explicitly compares the swga pipelines in 1.0 and 2.0 and shows the difference in the time needed to run each program. We have assisted several other labs in designing primer sets for their systems using swga2.0. However, we think it would not be appropriate to present those data in this manuscript. One collaborative project for which we used the swga2.0 pipeline to design a protocol to amplify Leishmania from both laboratory-created and real human samples has been submitted and is now referenced (`www.medrxiv.org/content/10.1101/2022.09.06.22279552v1`).

Major Comments:

1. Table 2: It is important to emphasize clearly that these features are specific for phi29. Some groups have begun using Equiphi29 to achieve higher reaction temperatures (enabling longer/more specific primers). Others may want to adapt these methods for other enzymes such as Bst. If the features employed by the program are phi29-specific, then the model will probably not perform well for these applications.

> **This is an important comment and we have now added a discussion of this topic. It is likely that most of the parameters in the random forest primer efficacy model will work for both phi29 and other polymerases (EquiPhi29 or Bst). Most of the parameters assess DNA-DNA binding potential and thus should be independent of the polymerase used. Other features are likely to be affected by the polymerase chosen, such as melting temperature. Conservatively, it would be prudent to empirically validate the primer efficacy model using EquiPhi29 or Bst.**

2. Page 4: It would be useful to delineate more clearly how swga2.0 differs from the original swga program published by Clarke et al., both in terms of the approach and empirical differences in output. As for approach, in the figure on page 4, there appear to be new steps in boxes 1 (jellyfish), 2 (homodimerization probability, runs of repeats), and most importantly in boxes 3 (random forest model) and 4 (coverage ratio). Some of these differences are covered on page 17 in the Discussion, but a table highlighting differences/improvements would be helpful.

> **Great idea. We have added Table 1 in the revised manuscript (and presented as Table 1 here).**

As for empirical differences in output, the authors include some discussion of differences on page 18 but are not comparing apples-to-apples. Differences between P. melaninogenica swga2.0 output and M. tuberculosis swga output are interesting, but direct comparison of swga vs swga2.0 for the same organism is needed to delineate their differences clearly. Did the authors run the original sWGA program on P. melaninogenica? If so, how did the output compare? If not, can they run it using similar parameters and comment on differences?

> **This was an omission of important information. We have now run both v1 and v2 on the same computer to directly compare the computational time saved and their outputs. It is difficult to make perfect comparisons due to the differences in parameters. Thus, we used the simplest (*i.e.* most time efficient) settings for swga1.0 in order to design primer sets for *Prevotella*. We compared this to the time it took to run swga2.0 using the settings we actually used to find the primer sets used in this manuscript. We have broken this down by stages in Table 1 (presented as Table 1 here). Overall, finding primer sets using swga2.0 is nearly 10 times faster than v1. More importantly, the outputs**

are fundamentally different as the majority (>80%) of candidate primers identified in swga1.0 are filtered by swga2.0. Further, all of the primer sets identified by swga1.0 are dominated by these low-amplification primers, regardless of the settings used, such that these sets are likely to perform poorly on the bench. Lastly, swga1.0 can search only a very limited number of the possible primer sets within a reasonable time such that many of the primer sets found by swga2.0 would not have been found by swga1.0 even when the same candidate primers are used.

3. How did variant calls in the P. melaninogenica sequencing data compare to reference and to the non-sWGA sample? Phi29 is reported to have a very low error rate, but it is important to confirm with their own data if possible.

This is a great idea. We have now included in Table S4 (see Table 4 below) which shows the number of single nucleotide differences between the reference genome sequence and our sequencing data pre-amplification ("Control") and for each of the post-amplification sets. The error rate was slightly lower for all of the post-amplification samples than for the pre-amplification control sample suggesting that phi29 does have a very low error rate.

Table 4: Proportion of called bases matching the *Prevotella* reference genome demonstrates that SWGA does not introduce sequencing errors.

Sample	Bases called	% matching reference
Control	2,430,966	99.9933%
Prev03A	2,932,347	99.9976%
Prev03B	2,987,130	99.9987%
Prev06A	3,048,879	99.9989%
Prev06B	3,119,187	99.9996%
Prev09A	1,642,904	99.9926%
Prev09B	2,635,407	99.9953%

Another issue of relevance in some cases is the ability to investigate within-host diversity after sWGA – specifically whether allele frequencies can still be estimated with confidence after sWGA enrichment (ex: Oyola et al. PMID 27998271). Though it is probably out of the scope of this manuscript to test experimentally, commenting on these effects in the discussion is probably worthwhile.

This is a good point that is worth discussing. We do not have empirical data to address this comment for the laboratory-made *Prevotella* samples. However, the biochemistry and types of primers are the same when working with swga1.0 and 2.0. Thus, we expect that researcher should be able to confidently estimate allele frequencies using the protocols designed with swga2.0.

4. Confidence in the program's ability to produce effective sWGA primer sets would be increased if more than one pathogen were tested experimentally. In the original swga publication, Clarke et al. reported differences in sWGA performance across several different organisms. I wonder if the new swga2.0 algorithm might overcome some of these differences, or if they will persist?

> **The new swga2.0 pipeline has been used to design successful protocols to amplify microbial species in several systems. One collaborative project that is nearing completion amplified *Leishmania* from both laboratory spike-in samples and from real patient samples. The data are not included in this manuscript but the project that produced those data is now referenced. We have also assisted other groups in designing primers using swga2.0 that were successful but those data have not yet been published.**

Minor Comments:

Page 2: sWGA has now been used successfully for Treponema pallidum subs pallidum (causative agent of syphilis) – see Thurlow CM et al. mSphere 2022, PMID 35491834.

> **Thank you. This has now been cited**

Page 3: Unclear why 'SWG amplification' is used instead of 'sWGA'.

> **This has now been fixed.**

Page 5: Please define each of the ratios included in the score equation listed at the bottom of the page.

> **This scoring function is now described in the section "Primer set scoring function" and in Table 4. We have also made the text more explicit no better address this topic.**

Pages 6-7 and Table 2: The included explicit discussion of thermodynamically-principled included in the model is helpful.

> **Thank you.**

Page 8: Why was the sample digested? I assume this is to linearize circular bacterial DNA. Has this been shown to improve the efficiency of MDA/phi29 reactions? In this paper, DNA was digested with FspEI while the previous study using NarI (Clarke et al., 2017). Methylation digestion with different enzymes and experimental process can vary the swga results, such as the proportion of reads mapping to the target organism and genome coverage (see Teyssier NB et al. PMID 33637093).

This is a good question that is a bit beyond the scope of this manuscript. Nevertheless, it is worth noting as several users are likely to have a similar question. We have now included a brief consideration of the use of restriction digests. Briefly, some prior protocols have digested the genomic DNA prior to amplification with some success, while others found no advantage (now cited). The idea of selective digestion is to reduce the length of background DNA which gives the target DNA an amplification advantage with phi29 (the length of template correlates with the amount of exponential amplification with phi29). However, we did try to amplify *Prevotella* using random heptamers from digested samples and gained only a minor target amplification advantage. These data suggest that digestion may not have been necessary in this system, although we did not assess this extensively.

Page 8: For clarity, please specify exactly what 3.5mM total of sWGA primers means so that there is no ambiguity. Ex: "…(e.g. in a set of 10 primers, each primer was added at 0.35mM so that the total molarity of primers was 3.5mM)" if that is the case, or other wording depending on the actual conditions. There has been variability in published sWGA methods on this topic, due in part to ambiguity in the description of the exact conditions used during sWGA in some past publications.

Good catch, thank you. You have put it correctly: in a set of 10 primers, each primer was added at 0.35mM so that the total molarity of primers was 3.5mM. We have made that more clear in the methods of the revised manuscript.

Page 18: The general suggestions provided are very helpful for scientists looking to use this methodology to design and test their own custom sets.

We hope so. Thank you.

Page 19: Mention of CRISPR and gRNA design in the concluding sentence doesn't follow from the manuscript and was surprising. Consider removing, or moving up into the discussion.

We agree that this was speculative. We have removed this paragraph as we do not have the empirical data to support it.

Methods: In swga2.0, is it possible to visualize/export exact locations of primer binding sites?

The exact primer locations can be visualized in bedgraph and other available software. However, swga2.0 also explores amplification from primer binding sites that are not exact matches, which would not be as easily visualized.

Methods: Is it possible to set the number of mismatched bases permitted in the primers themselves? Can the allowable length of mononucleotide repeats be modified?

These are not currently features in the program. The current algorithm uses a data-driven, machine learning approach that identifies the optimal parameters for these filters. However, the set of candidate primers at the end of stages 2 and 3 are saved in .csv file, and these can be very easily processed with any additional custom filters a user would like.

Have the authors made all data and (if applicable) computational code underlying the findings in their manuscript fully available? The PLOS Data policy requires authors to make all data and code underlying the findings described in their manuscript fully available without restriction, with rare exception (please refer to the Data Availability Statement in the manuscript PDF file). The data and code should be provided as part of the manuscript or its supporting information, or deposited to a public repository. For example, in addition to summary statistics, the data points behind means, medians and variance measures should be available. If there are restrictions on publicly sharing data or code —e.g. participant privacy or use of data from a third party—those must be specified.

Reviewer 1: No: The code is available and well documented but I do not see the sequencing reads, the raw data, or the primer sequences.

We apologize for this oversite. The primer amplification data are now Table S1, the primer sequences and associated statistics are Table S2, and the sequencing reads have been uploaded to Dryad (`https://doi.org/10.5061/dryad.3n5tb2rm2`).

Reviewer 2: Yes
