# Supplementary material for: A fast machine-learning-guided primer design pipeline for selective whole genome amplification
Source: PLoS Comput Biol. 2023 Apr 17;19(4):e1010137. doi: 10.1371/journal.pcbi.1010137 (PMC10138271; doi:10.1371/journal.pcbi.1010137)
Supplement: Attachment — Submitted filename: Response_To_Reviewers.pdf [file pcbi.1010137.s007.pdf]

Response to reviewers

We thank the editors and reviewers for their detailed and constructive assessment of our manuscript. The updated manuscript addresses all recommendations as suggested by each reviewer. To distinguish our responses from the original reviewer comments, we have labeled reviewer comments in **blue** and our responses in **black**.

Reviewer 1: This resubmission is much improved, and the authors have addressed my major concerns. I have a few minor comments.

> **Thank you. We hope it is useful to the community.**

The authors added Table 1 which helpfully summarizes the improvements in swga2.0 compared to swga1.0 and breaks down the performance improvements for all stages. However, the benchmarking details could be a bit more explicit in a computational biology article. For example, I had to go to the Github page to find swga2.0's default setting for number of CPUs (which I assume is what was used). After finding out that it defaults to "all" I then needed to look up what that might be on the specific model of MacBook Pro that was used (4 maybe)? I think that this is important information to have in the article to evaluate the 10-fold improvement in speed. Another minor point on Table 1, the first column could include a short name/description of each of the stages in addition to just the stage number so that the reader does not have to refer to the text to understand the table.

> **We agree with these recommendations and have updated Table 1 to include the specific MacBook Pro model used in these experiments and included short, descriptive names for each stage. Additional default parameter values for swga2.0 are now included in the manuscript.**

My previous comment about the method for calculating amplification efficiency was referring to the empirical values, not the predictions using the model. Some detail about the method used to measured primer amplification of the plasmids would be helpful, even if it is only included in the caption for Supplemental Table 1.

> **We have now included a brief description of the methods used to measure primer amplification success from plasmids to the captions of Supplemental Table S1.**

All other issues that I have raised have been addressed by the authors.

> **Thank you for the detailed assessment that has improved this manuscript.**

Reviewer 1: I appreciate the authors' responses to my comments and the associated manuscript changes. One minor comment as below:

Discussion: The authors include the following statement: "Also similar to prior results (Oyola et al., 2016), protocols developed with swga2.0 retain the ability to investigate within-host microbial diversity using SWGA enrichment (Pilling et al., 2022), without introducing errors, as the SWGA biochemistry remains identical (Table S4)."

We have found that reliable estimation of allele frequencies after sWGA is not straightforward, due in part to exceptionally high depth of coverage in some regions ("jackpotting") but not others. If the authors do not have empirical data to support the statement above, I would suggest softening the language to indicate that this is their expectation but not an observation (i.e. replace "retain" and with "are expected" or similar).

We have change the language of this statement as suggested by the reviewer.
